# Biogeographic patterns of bacterial microdiversity in Arctic deep-sea sediments (HAUSGARTEN, Fram Strait)

**DOI:** 10.3389/fmicb.2014.00660

**Published:** 2015-01-05

**Authors:** Pier Luigi Buttigieg, Alban Ramette

**Affiliations:** ^1^Hinrichs Lab, Organic Geochemistry Department, MARUM – Center for Marine Environmental SciencesBremen, Germany; ^2^HGF-MPG Bridge-Group for Deep Sea Ecology and Technology, Alfred-Wegener-Institut, Helmholtz-Zentrum für Polar- und MeeresforschungBremerhaven, Germany; ^3^Max Planck Institute for Marine Microbiology, HGF-MPG Bridge-Group for Deep Sea Ecology and TechnologyBremen, Germany

**Keywords:** HAUSGARTEN, oligotyping, deep sea sediments, Arctic LTER, taxonomic resolution

## Abstract

Marine bacteria colonizing deep-sea sediments beneath the Arctic ocean, a rapidly changing ecosystem, have been shown to exhibit significant biogeographic patterns along transects spanning tens of kilometers and across water depths of several thousand meters (Jacob et al., 2013). Jacob et al. (2013) adopted what has become a classical view of microbial diversity – based on operational taxonomic units clustered at the 97% sequence identity level of the 16S rRNA gene – and observed a very large microbial community replacement at the HAUSGARTEN Long Term Ecological Research station (Eastern Fram Strait). Here, we revisited these data using the oligotyping approach and aimed to obtain new insight into ecological and biogeographic patterns associated with bacterial microdiversity in marine sediments. We also assessed the level of concordance of these insights with previously obtained results. Variation in oligotype dispersal range, relative abundance, co-occurrence, and taxonomic identity were related to environmental parameters such as water depth, biomass, and sedimentary pigment concentration. This study assesses ecological implications of the new microdiversity-based technique using a well-characterized dataset of high relevance for global change biology.

## INTRODUCTION

Ecological analyses are typically concerned with gauging the response of a collection of organisms, grouped into coherent units such as species, to the biotic and abiotic factors affecting them. Establishing meaningful units of bacterial diversity is an ongoing challenge in the microbial sciences (Cohan, 2001, 2002; Kopac and Cohan, 2011; McDonald et al., 2013; Mende et al., 2013) and the nature of these units has been shown to strongly influence the outcomes of ecological analyses (see e.g., Koeppel and Wu, 2014). An approach that has become a standard in microbial ecology relies on the classification of organisms into units based on the level of sequence identity between their 16S rRNA genes. At the more granular end of this classification, organisms that have 16S rRNA gene sequences that are at least 97–98% identical are grouped into operational taxonomic units (OTUs) which are treated as approximations of bacterial ‘species’ in further analyses. However, it has been shown that the organisms grouped into a single OTU, at times with identical 16S sequences, can show ecologically meaningful genetic and physiological differences, allowing them to colonize distinct niches (e.g., Moore et al., 1998; Hahn and Pöckl, 2005; Coleman et al., 2006).

While alternative differentiae must be sought for organisms with identical 16S genes, the entropy-based method of “oligotyping” (Eren et al., 2013; not to be confused with oligotyping *sensu* Tiercy et al., 1990) offers an approachable means to detect whether position-specific, subtle sequence variation at up to single-nucleotide resolution can reveal coherent, sub-OTU groupings with differential occurrence across samples or responses to environmental factors. This technique has been applied in investigations of human-associated microbes, such as those that compose the oral (Eren et al., 2014a) and gut (Eren et al., 2014b) microbiomes, as well as of aquatic (Eren et al., 2013) and wastewater environments (McLellan et al., 2013), and in the assessment of *Gardnerella vaginalis* diversity (Eren et al., 2011). Such studies have revealed that subtle nucleotide variations can, reproducibly, be associated with distinct environments, hosts, or epidemiological states and encourage the exploration of oligotype-based microdiversity in similar sequenced-based datasets.

Here, we employed oligotyping to reanalyze data from a previous investigation (Jacob et al., 2013) which assessed biogeographic patterns of deep-sea, benthic bacterial diversity at the Long Term Ecological Research (LTER) station, HAUSGARTEN in the Eastern Fram strait (Soltwedel et al., 2005). This LTER comprises two transects, one bathymetric (water depths between ~1000 and ~5500 m) and one latitudinal (at a depth of ~2500 m), intersecting at a central site. At this station, heat- and nutrient-laden Atlantic waters carried by the West Spitsbergen Current flow northward into the Arctic, separated from the cold Eastern Greenland Current by the East Greenland Polar Front. When present, sea ice attenuates light input and, hence, under-ice primary productivity; however, phytoplankton blooms and phytodetritus pulses occur along melting ice-edges where primary producer communities in the ice are released into the irradiated and meltwater-stabilized water column (Schewe and Soltwedel, 2003; Leu et al., 2011; Boetius et al., 2013). The organic and inorganic detritus supplied to the benthos is of varying composition, either produced in the photic zone of the water column or transported by physical processes such as advection or sea ice rafting (Hebbeln, 2000; Bauerfeind et al., 2009). Due to remineralization processes in the water column, phytodetritus availability decreases with increasing water depth, producing a depth-related gradient in this key component of benthic food supply. Within this system, prokaryotic communities are responsible for over 90% of the respiration performed in a food web sensitive to changes in labile detritus input (van Oevelen et al., 2011). In recent years, notable changes in the system’s oceanography, biogeochemistry, and biology have been reported. For example, anomalously warm Atlantic inflows from 2005 to 2007 impacted the composition of the detritus exported to the benthos: reduced export of particulate carbon, zooplankton fecal pellet carbon, and biogenic silica suggested a shift in the composition of phytoplankton communities to favor small, non-siliceous organisms (Piechura and Walczowski, 2009; Lalande et al., 2013). Additionally, changes in Arctic ice dynamics and the loss of multi-year ice – along with its resident, ice-associated communities – are expected to impact biological input to this system, reducing benthic–pelagic coupling (Hop et al., 2006) as observed in other regions of the Arctic (Grebmeier et al., 2006).

Within this context, Jacob et al. (2013) sampled undisturbed sediments along the HAUSGARTEN bathymetric transect (HGI-HGVI; with a depth range of 1284–3535 m along 54 km) and latitudinal transect (N1–N4, HGIV, and S1–S3; 78.608–79.717 N, at a depth of ~2500 m along 123 km) during July 2009. The authors examined bacterial communities present in the oxic, upper centimeter of the sediment surface. The authors clustered sequences of the 16S rRNA gene’s V4–V6 region into OTUs at the conventional sequence identity threshold of 97%. They then derived matrices of OTU relative abundances at each site. Jacob et al. (2013) investigated the response of bacterial diversity, community structure, and spatial turnover across taxonomic levels and found water depth to be a central explanatory parameter, in line with findings on a global scale (Zinger et al., 2011) and in other regions of the Arctic (Bienhold et al., 2012). To assess if subtle nucleotide variation can reveal finer-grained variation in this data, we oligotyped several, abundant OTUs detected in the Jacob et al. (2013) study and (1) examined the degree of separation and/or aggregation of intra-OTU oligotypes across sites, (2) assessed the influence of environmental and spatial variables on oligotype variation, and (3) examined the composition and structure of oligotype association networks, inferred by co-occurrence across both transects. Through these analyses, we aimed to explore oligotyping’s potential as a means to enhance the characterization of bacterial diversity at HAUSGARTEN.

## MATERIALS AND METHODS

### SEQUENCE DATA PROCESSING AND OLIGOTYPING

Sequences obtained by 454 pyrosequencing of the 16S rRNA gene’s V4–V6 region (*n* = 145,938) were previously trimmed and denoised by Jacob et al. (2013) using *mothur* (Schloss et al., 2009). We submitted these trimmed and denoised sequences to the SILVAngs pipeline (v1.0; Quast et al., 2013) using the pipeline’s default parameters – save for an OTU clustering threshold of 97% sequence identity – and quality filtering measures. As pyrosequencing-derived reads of varying length were used in this study, alignments were performed by the SILVA incremental aligner (SINA v1.2.10 for ARB SVN [revision 21008]; Pruesse et al., 2012) and OTU classification was performed against the SILVA SSU Ref dataset (release 115). Alignments were examined and terminal regions with poor coverage trimmed in the ARB environment (Ludwig et al., 2004); however, some positions with incomplete but good coverage over all alignment positions were retained. In doing so, we reasoned that if the alignment was to be split among oligotypes in such a way that only valid sequence data was present at a globally incomplete but well-covered position, that position would be a valid target for oligotyping. However, if a resulting oligotype was derived from an incomplete alignment, it was removed from further analysis. The resulting alignments were exported for oligotyping.

Reads belonging to OTUs with total read counts greater than 100 were oligotyped (Eren et al., 2013) to convergence by recursively selecting the alignment position(s) with the greatest entropy for each round of oligotyping. At each step, a round of oligotyping was only performed on alignments which featured at least 21 sequences and included a position with entropy greater than 0.6 (see **Table 1** and Discussion). The oligotyping output was not restricted by any of the software’s command line parameters such as the minimum percent, actual, or substantive abundance. Output from the oligotyping software and SILVAngs pipeline were and then imported into the R environment (R Development Core Team, 2014) for further processing and analysis.

**Table 1:** Entropy in terms of the proportion of deviations from the expected character in a character sequence and the percentage of the dominant character in that sequence.

Entropy	Proportion of alternate characters relative to the dominant character present at an alignment position	Percent occurrence of the dominant character present at an alignment position
0.65	1:5	83.3
0.60	1:6	85.7
0.44	1:10	90.9
0.28	1:20	95.2
0.21	1:30	96.8
0.14	1:50	98.0
0.08	1:100	99.0
0.02	1:500	99.8
0.01	1:1000	99.9

### DATA PREPARATION

Geographic coordinates were converted from Global Positioning System (GPS) coordinates to Universal Transverse Mercator (UTM) coordinates (i.e., Easting and Northing in m) using the *sp* (Pebesma and Bivand, 2005) and *rgdal* (Bivand et al., 2013) R packages. Further, all count data were Hellinger transformed prior to applying redundancy analysis (RDA). Environmental variables, comprising pigment, protein, and phospholipid concentrations as well as spatial variables (Easting, Northing, and water depth) were z-scored (i.e., set to zero mean and unit variance).

### GENERAL EXPLORATIONS

**FIGURE 1:**
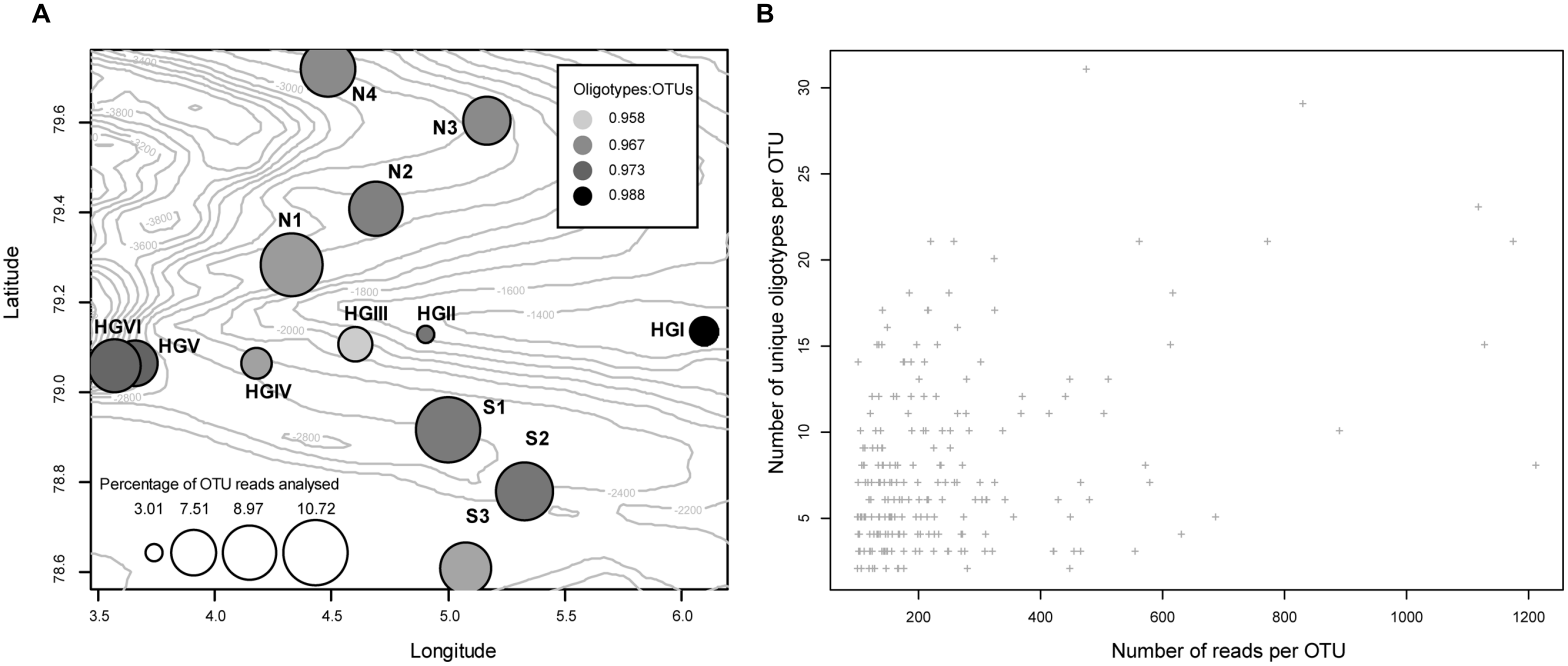
**(A)** Bubble plot approximating the location of each sampling site (bubble coordinates), the per site, percentage contribution of reads used in this study (bubble size), and the sample-specific ratio of the number of reads that were present in an oligotype to the number of reads in the OTU it was derived from (fill intensity). Numeric values on isobaths indicate the depth of the seafloor in meters below the water surface. **(B)** The total number of reads clustered in a given OTU plotted against the number of oligotypes derived from that OTU (Pearson’s *R*^2^ = 0.39, *P* <<0.01).

**FIGURE 2:**
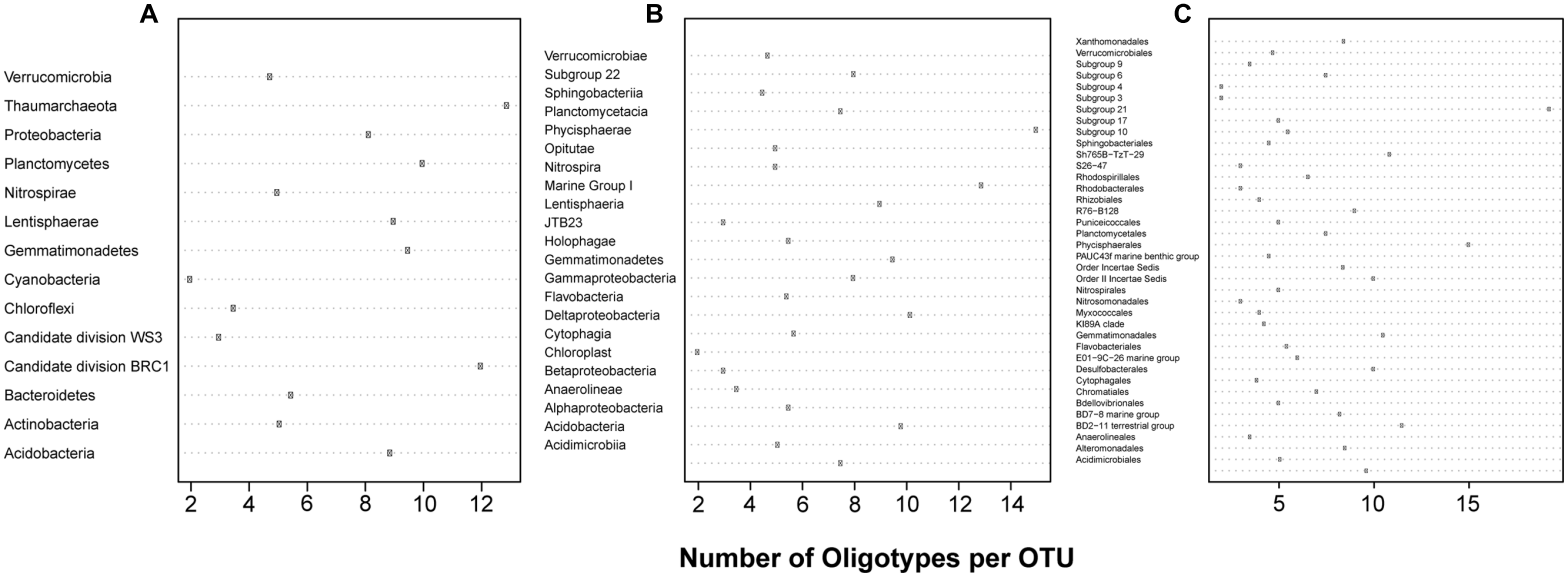
**The oligotype:OTU ratio for each (A) Phylum (B) Class and (C) Order analyzed**.

**FIGURE 3:**
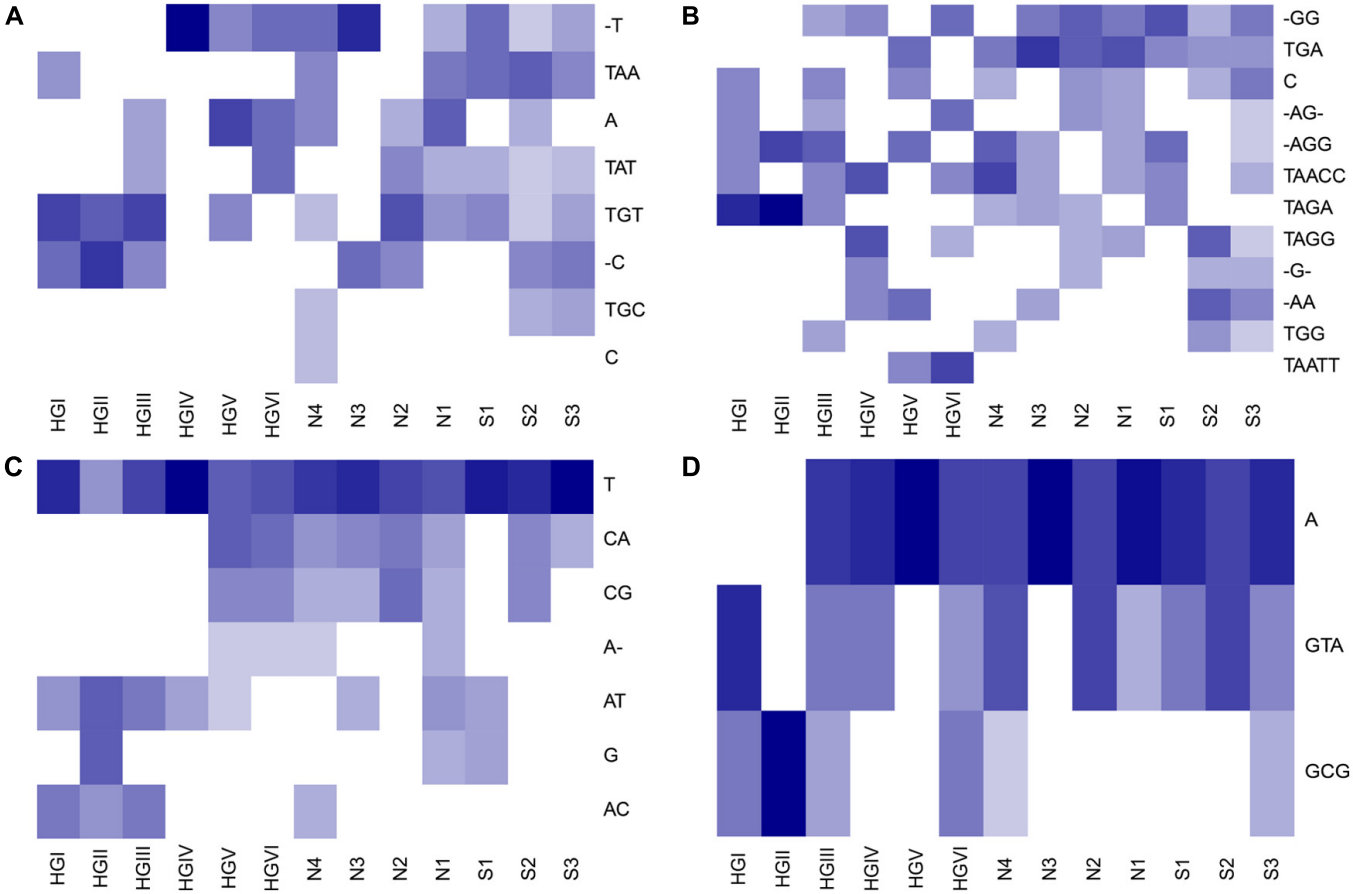
**Heatmaps illustrating examples of abundance matrices subject to checkerboard and togetherness score screening.** Rows (oligotypes) have been Hellinger transformed and ordered by hierarchical cluster analysis using average linkage and Bray–Curtis dissimilarities. Darker shades indicate higher relative abundance of reads. In the following text, the maximum, untransformed number of oligotype reads in each OTU-derived relative abundance matrix is noted in brackets. The oligotypes for OTUs **(A)** EUGQ5 [8], which had the joint-highest average T score observed; **(B)** B177D [5], which had both the highest average C and T scores observed; **(C)** C60MC [12]; and **(D)** DON2B [9] which both had high average C and T scores are displayed (cf. **Table 2**). AGCT: nucleotides; -: gap.

Simple diagnostic plots were created to (1) illustrate each sampling location’s percent contribution of reads to this analysis and illustrate the per location percentage of reads retained (relative to the reads present in all OTUs at that location) following removal of those reads belonging to oligotypes with incomplete alignments (**Figure 1A**), (2) compare the number of reads clustered in a given OTU to the number of unique oligotypes derived from it (**Figure 1B**), and (3) visualize the proportion of oligotypes derived from OTUs across specific higher-order taxa (**Figure 2**).

### DETECTING ‘RESOLVING’ OLIGOTYPES

For each OTU selected for analysis, we calculated the mean “checkerboard” (C) and “togetherness” (T) scores (Stone and Roberts, 1992) of its oligotypes using the R package bipartite (Dormann et al., 2009). High C scores indicate that pairs of oligotypes occur in checkered patterns across samples. That is, one oligotype’s presence and absence is repeatedly mirrored by another’s in two-by-two units, resembling a similarly sized unit of a checkerboard. High T scores indicate that pairs of oligotypes tend to occur in aggregates across samples, being simultaneously present or absent. Both C and T scores can be high (relative to those calculated from a random distribution of presences and absences) should groups of aggregated oligotypes, the existence of which will increase the average T score of a matrix, form checkered patterns with other groups, increasing the average C score. Based on these distributions, we selected oligotypes with average checkerboard and togetherness scores greater than the third quartile of all scores measured for further investigation. These oligotypes were treated as candidate ‘resolving’ oligotypes. A resolving oligotype would thus be heterogeneously distributed across sites, but would cluster with other, similarly distributed oligotypes. Hellinger-transformed abundance matrices were visualized as heatmaps with oligotypes grouped by hierarchical cluster analysis (using average linkage) of the corresponding Bray–Curtis dissimilarity matrices.

### DETECTING ENVIRONMENTALLY STRUCTURED OLIGOTYPES

We applied RDA as implemented in the R package *vegan* (Oksanen et al., 2013) to Hellinger-transformed oligotype abundance matrices derived from each oligotyped OTU. Forward selection, as described by Blanchet et al. (2008), was used to select explanatory variables across all RDA solutions calculated. The full model’s explanatory matrix comprised the following variables: particulate protein concentration, pigment concentration (CPE), Easting, Northing, and water depth. Models associated with a percentage of constrained variation greater than 50% and *P*-values less than 0.05 were investigated further. All *P*-values were corrected for multiple testing using the base R function, p.adjust, employing the method of Benjamini and Hochberg (1995). Variance inflation factors (estimated with *vegan’s* vif.cca function) were verified to be <10 to ensure constraints were not multicollinear.

### EXPLORING OLIGOTYPE ASSOCIATIONS

Associations between oligotypes were explored using graph theoretic approaches. Only those oligotypes with a total relative abundance greater than one were considered. A graph was created with oligotypes as nodes, and edges defined by the value of Whittaker’s index of association (IA), as described by Somerfield and Clarke (2013), calculated for each pair of oligotypes. This index is similar to the one-complement of the well-known, asymmetric Bray–Curtis dissimilarity; however, variable (i.e., oligotype) proportions are scaled such that they sum to 100. Consequently, oligotypes with identical percentage abundances across samples have an IA of 100, while those that with no overlapping occurrence across samples have an IA of zero. Significance was assessed by independently permuting (*n* = 200) the sample order in each oligotype abundance vector of the original dataset and recalculating a matrix of IA values. The probabilities of the observed IA values given the permuted values were corrected for multiple testing using the method of Benjamini and Hochberg (1995).

Oligotypes with an IA greater than 85 and an FDR-corrected *P*-value less than 0.05 were linked by an edge and the corresponding IA value was used as an edge weight. The Cytoscape suite (v 3.1.1; Smoot et al., 2011) was used to visualize and analyze the graph object. Node size was scaled by the total abundance of each oligotype (minimum = 2, maximum = 330) and edge width by the value of its weight. The Markov cluster (MCL) algorithm (Enright et al., 2002), as implemented in the *clusterMaker 2* (Morris et al., 2011) Cytoscape ‘app,’ was used with its default granularity parameter value of 2.5 to identify clusters. As recommended by van Dongen and Abreu-Goodger (2012), the edge weight interval was adjusted from 0.85–1 to 0.001–0.15 to allow better performance of the MCL algorithm.

## RESULTS

A total of 19,283 OTUs were generated by the SILVAngs pipeline, of which 95.86% were taxonomically classified. Of these, 217 were represented by at least 100 reads, passed our thresholds for oligotyping, and were used in further analysis. Despite this study targeting bacterial organisms, eight OTUs classified as Thaumarchaeota (Marine Group I) were included in further analyses. Following the oligotyping procedure described above, 1,694 oligotypes were identified, 290 of which were singletons. The minimum, median, and maximum numbers of oligotypes per OTU were 2, 6, and 31, respectively. The oligotyped OTUs represented 14 Phyla, 23 Classes, and 29 Orders (**Figure 2**).

### WITHIN-OTU OLIGOTYPE ABUNDANCES SHOW VARIATION ACROSS SAMPLES

Oligotype matrices derived from a total of 25 OTUs possessed average C and T scores above the third quartile of these measures as distributed across all 217 oligotype matrices calculated (i.e., >3.60 and >6.17, respectively; **Table 2**). These scores showed no notable correlation (Pearson’s *R*^2^ = ~0.25, *P* = 0.22). The majority of these OTUs were classified as Acidobacteria or Proteobacteria; however, the highest average C scores belonged to oligotypes of reads assigned to the phyla Gemmatimonadetes and Bacteroidetes, as well as the Candidate division WS3. The highest T scores were observed for reads assigned to the Acidobacteria, Proteobacteria, and Gemmatimonadetes. To illustrate the patterns associated with these average measures, several Hellinger-transformed oligotype abundances were visualized as heatmaps in **Figure 3**.

**Table 2:** Average checkerboard and togetherness scores for oligotype occurrence matrices generated from selected OTUs, cf. to **Figure 3**.

OTU ID	Phylum	Class	*n* oligotypes	Mean C score	Mean T score
A83S4	Acidobacteria	Acidobacteria	17	5.16	7.63
DF5XB	Acidobacteria	Acidobacteria	15	4.70	7.01
AS91F	Acidobacteria	Acidobacteria	10	4.60	6.29
EDBYN	Acidobacteria	Subgroup 22	7	5.95	7.19
BTL2B	Acidobacteria	Subgroup 22	9	3.89	9.31
CEL9R	Actinobacteria	Acidimicrobiia	8	4.11	6.18
BRUV2	Actinobacteria	Acidimicrobiia	11	5.98	6.36
DD9DS	Actinobacteria	Acidimicrobiia	6	4.73	9.00
C60MC	Bacteroidetes	Flavobacteria	7	7.05	7.00
DON2B	Candidate division WS3	–	3	7.00	6.33
B177D	Gemmatimonadetes	Gemmatimonadetes	12	8.76	9.39
A5C8S	Planctomycetes	Planctomycetacia	8	4.71	7.21
EMCAY	Proteobacteria	Alphaproteobacteria	6	5.67	7.13
EAFF9	Proteobacteria	Alphaproteobacteria	10	3.71	7.00
BQX8G	Proteobacteria	Deltaproteobacteria	12	6.06	8.02
CMQFL	Proteobacteria	Deltaproteobacteria	15	3.90	6.29
DS0T4	Proteobacteria	Deltaproteobacteria	12	6.30	8.44
DSTJG	Proteobacteria	Deltaproteobacteria	7	5.00	6.76
CA3XY	Proteobacteria	Gammaproteobacteria	7	3.71	7.19
EUGQ5	Proteobacteria	Gammaproteobacteria	8	5.57	9.39
AESJT	Proteobacteria	Gammaproteobacteria	14	6.36	7.65
BQD17	Proteobacteria	Gammaproteobacteria	10	4.60	6.62
AJ3H1	Proteobacteria	Gammaproteobacteria	18	5.90	6.24
EBMBR	Proteobacteria	Gammaproteobacteria	15	5.47	6.49
E00H7	Verrucomicrobia	Verrucomicrobiae	9	4.72	6.64

		**Average**	~ **10**	**5.34**	**7.31**

### ASSESSING ENVIRONMENTAL AND SPATIAL EFFECTS ON OLIGOTYPE ABUNDANCES

**FIGURE 4:**
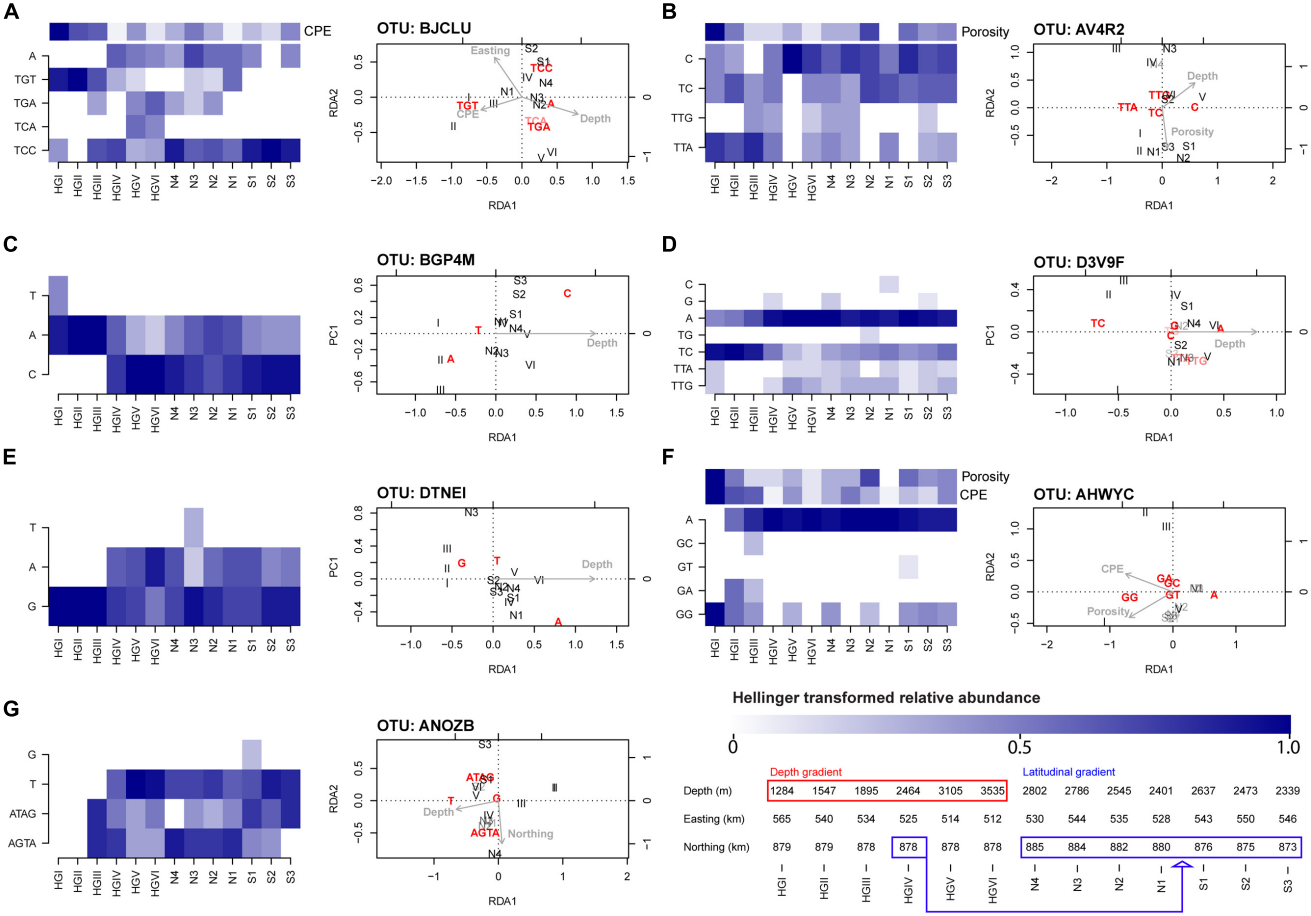
**Heatmaps and RDA triplots (type 2 scaling) derived from Hellinger-standardized, OTU-specific oligotype relative abundance matrices.** The seven models shown had at least 50% constrained variance and were significant at a *P*-value threshold of 0.05 (FDR-corrected) cf. **Table 3**. The depth and latitudinal gradients sampled are reflected in the sample order (HGI shallowest, HGVI deepest, N4 northernmost, S3 southernmost) and elaborated upon in the bottom right of the figure where the depth and latitudinal gradients are highlighted with red and blue boxes, respectively. Site HGIV, the central site of the intersecting transects, belongs to both transects. See **Figure 1A** Jacob et al. (2013) for greater detail. When other explanatory variables were featured in the model, an additional heatmap of these variables’ z-scored values is included in the panel. Across heatmaps, darker shades indicate higher, Hellinger-transformed relative abundance of reads or higher values of a given explanatory variable. The panels reference oligotype abundance matrices derived from the following OTUs (the maximum, untransformed read abundance across oligotypes in each matrix is noted in brackets): **(A)** BJCLU [18] **(B)** AV4R2 [14] **(C)** BGP4M [16] **(D)** D3V9F [62] **(E)** DTNEI [19] **(F)** AHWYC [39] and **(G)** ANOZB [9]. Explanatory variables are represented by gray text and arrows pointing in their direction of increase, these comprise Porosity (range: 51.8–72.3% volume), CPE (18.86–44.26 μg cm^-3^), Northing (8727035–8850377 m), Easting (512100–565125 m), and Depth (1284–3535 m). Oligotypes (response variables) are ordinated as bold, red text. Relative to each plot’s origin, the position of an oligotype’s ordination indicates its direction of increase. Angles between variables indicate their linear correlation, with an angle of 0° indicating perfect positive correlation, 180° indicating perfect negative correlation, and 90° indicating orthogonality. Samples are ordinated as black text. Transparency effects are used to improve visibility in congested regions of the triplots and have no meaning.

After performing RDA combined with forward selection, we identified seven OTU-specific oligotype abundance matrices which had greater than 50% of their variation constrained by one or more explanatory variables (**Table 3**). All but one (AHWYC, of the Gammaproteobacteria) featured water depth as an explanatory variable, while porosity, CPE, and a spatial variable were each featured in two models. The triplots of these models, as well as corresponding heatmaps of their Hellinger-transformed oligotype abundances, are displayed in **Figure 4**. Oligotypes, ordinated as bold, red text, show differing responses to the selected explanatory variables. For example, the TGT oligotype of OTU BJCLU (**Figure 4A**) appears in higher relative abundances at shallower sites with higher CPE concentrations while the A oligotype of OTU DTNEI (**Figure 4E**) tends to increase in abundance at increased depth.

**Table 3:** Results of RDA on oligotype abundance matrices derived from selected OTUs.

OTU ID	Class	Order	*n* oligotypes	Model	Constrained variation (%)
BJCLU	Deltaproteobacteria	Bdellovibrionales	5	Y ~ Easting + CPE + Depth	69
AV4R2	Gammaproteobacteria	Incertae Sedis	4	Y ~ Depth + Porosity	52
BGP4M	Cytophagia	Cytophagales	3	Y ~ Depth	70
D3V9F	Gammaproteobacteria	Xanthomonadales	7	Y ~ Depth	69
DTNEI	Cytophagia	Cytophagales	3	Y ~ Depth	65
AHWYC	Gammaproteobacteria	Xanthomonadales	5	Y ~ Porosity + CPE	65
ANOZB	Cytophagia	Cytophagales	4	Y ~ Depth + Northing	60

*Explanatory variables were chosen through forward selection. All models had FDR-corrected *P*-values of, at most, 0.0098. Y: the response matrix of oligotype relative abundances; CPE: Pigment concentration; Depth: Water depth.*

### EVALUATING OLIGOTYPE-TO-OLIGOTYPE ASSOCIATION

**FIGURE 5:**
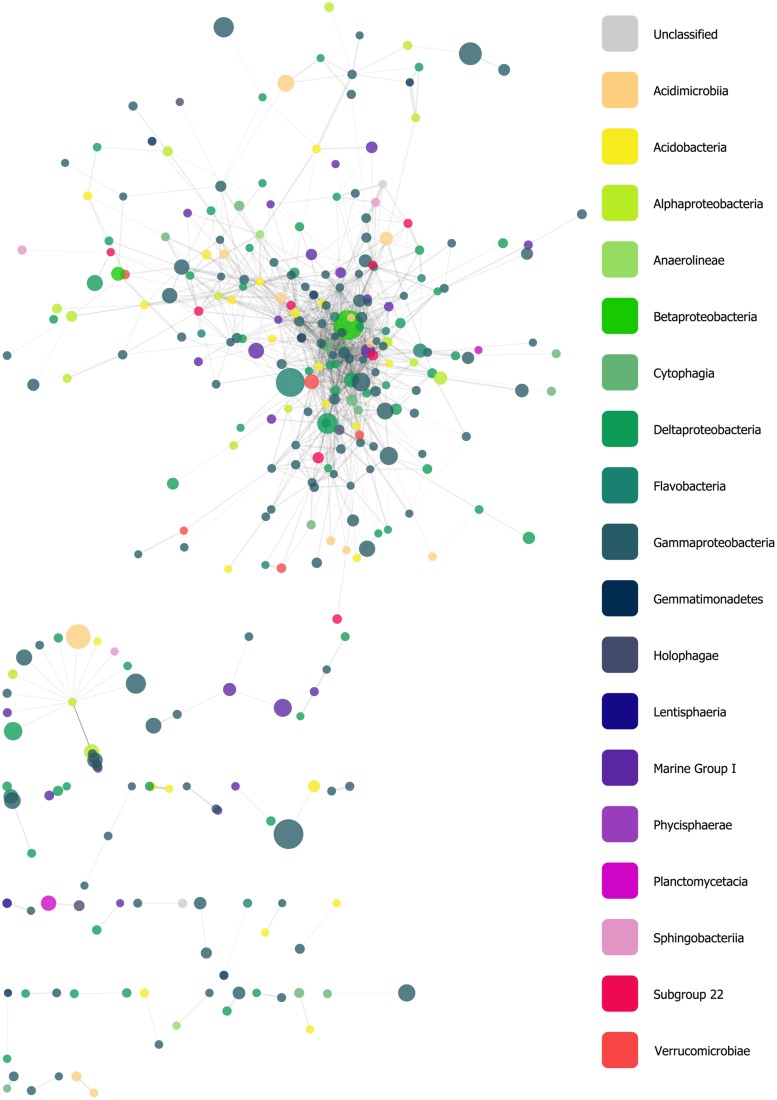
**Force-directed, spring-embedded network displaying oligotypes (nodes) with Whittaker’s index of association (IA) values greater than 85 (FDR-corrected *P*-values as determined by 200 permutations <0.05), represented as edges.** Nodes are color-coded by taxonomic Class. See text for a summary of this network’s general statistics.

We constructed a network derived from a filtered similarity matrix calculated using Whittaker’s IA (see Materials and Methods) which contained 318 nodes (oligotypes) and 1,308 edges (associations; **Figure 5**). A total of 32 connected components (CCs) of varying taxonomic composition were present; however, the network was dominated by a single CC with 225 nodes, while other CCs had between 22 and 2 nodes each. The network had a clustering coefficient of ~0.28, a density value of ~0.03, a heterogeneity value of ~1.33, a centralization value of ~0.16. Nodes had, on average, ~8.23 neighbors. Within the largest CC, these values were approximately 0.39, 0.05, 1.07, 0.21, and 11.08, respectively. Additionally the largest CC had scale-free properties with a degree-distribution following a power law: *y* = 72.6 × *x*^-1.1^. Node degree (i.e., the number of edges associated with a given node) ranged from 57 to 1. Of the 10 nodes with the highest degrees (between 39 and 57), five were classified in the Order Gammaproteobacteria (Family: Xanthomonadales), three as Cytophagia, and the remaining two were classified as a Deltaproteobacterium and an Acidobacterium with read abundances between 2 and 68.

**FIGURE 6:**
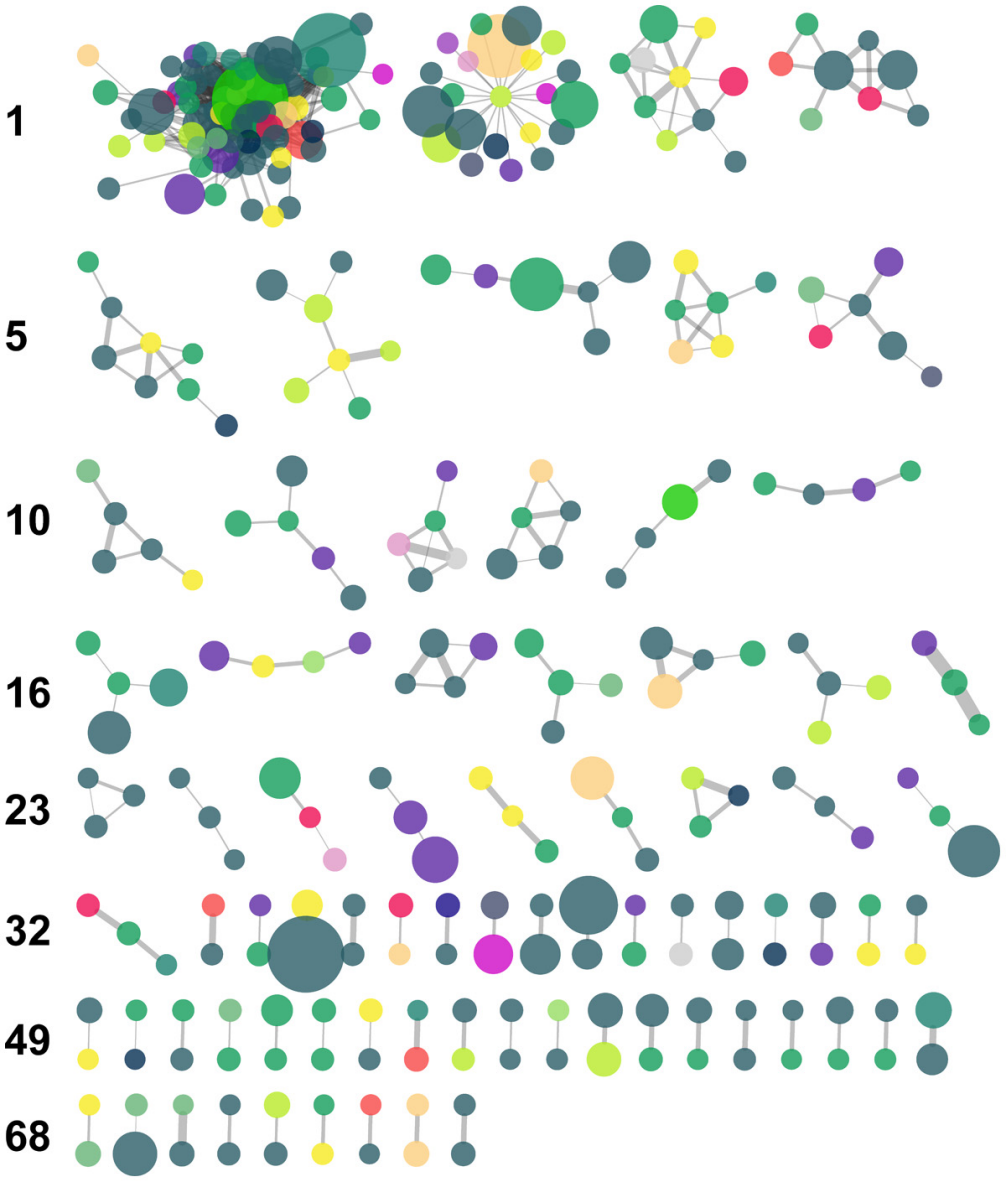
**Results of Markov clustering of the graph displayed in **Figure 5**, with a granularity parameter value of 2.5.** Nodes are color-coded by taxonomic Class (see **Figure 5** for key) and their size is proportional to the total relative abundance of the corresponding oligotype. Edge thickness is proportional to the value of the IA between oligotypes. The leftmost cluster of each row is numbered along the left margin.

The MCL algorithm generated 76 clusters of nodes which included oligotypes belonging to an assortment of taxa and with varying degrees of read abundance (**Figure 6** and **Table 4**). This algorithm resolved the largest CC into several clusters, the largest of which included 72 nodes.

**Table 4:** Selected characteristics of the five largest MC clusters with reference to the IA network cf. **Figure 6**.

Cluster	No. of oligotypes	Average and range of oligotype abundance	No. of phyla represented	No. of classes represented
1	72	36.2, 327	8	14
2	22	53.6, 250	6	11
3	10	23.7, 102	3	5
4	8	38.38, 111	4	5
5	8	10.5, 23	3	4

## DISCUSSION

In this study, we applied oligotyping to extant sequence data obtained from a unique and dynamic Arctic, deep-sea LTER. While our analyses were primarily exploratory, they indicate that subtle nucleotide variation does indeed provide a new perspective on bacterial diversity at HAUSGARTEN that is not redundant with that derived from OTU-based diversity data. Further, in observing that several of the oligotype abundance matrices derived from specific OTUs appear to be structured by environmental or spatial variables (**Table 3** and **Figure 4**), we are encouraged that further application of this technique – particularly in the context of ‘omic-centered,’ long-term research (see e.g., Davies et al., 2014) – will enhance the likelihood of identifying ecologically meaningful divergence at up to single-base resolution. This, in turn, may aid in the detection of ecotypes (e.g., Moore et al., 1998; Garczarek et al., 2007; Ivars-Martinez et al., 2008) and the concomitant deepening of knowledge surrounding the ecosystems they inhabit. Naturally, the success of such a strategy is directly determined by the selection of an appropriate genetic element, as the 16S gene may, in some cases, have poor resolving power (e.g., Jaspers and Overmann, 2004) and other markers may offer more scope (Lerat et al., 2003; Yilmaz et al., 2011; Mende et al., 2013).

### THRESHOLDS FOR OLIGOTYPE DETECTION

In the present case, we limited our analysis to OTUs with high read abundance in order to operate on relatively large alignments which could undergo several rounds of oligotyping. Under this constraint, we noted that the abundance of reads belonging to an OTU does not meaningfully correlate with the number of oligotypes it will be resolved into (**Figure 1B**), which reinforces the notion that understanding nucleotide variation is likely to require specific knowledge of the organisms, evolutionary characteristics, and ecology involved in the diversification processes at work (McDonald et al., 2013). We acknowledge that limiting our analysis to these abundant OTUs precludes the observation of many, potentially important oligotypes; however, we find it prudent to reserve more thorough analysis until a greater body of longitudinal sequence data is amassed at HAUSGARTEN. Repeated observation of oligotypes over time and the evaluation of their variation in the face of environmental variation will provide a far better basis for interpretation.

In addition to focusing solely on abundant OTUs, we only performed a round of oligotyping if an alignment with at least 21 sequences was available and entropy analysis revealed positions with entropy values greater than or equal to 0.6. We acknowledge that our choice of entropy and sequence count thresholds is, ultimately, arbitrary. **Table 1** partly clarifies the nature of our selection: with an entropy value of 0.6, one can expect 85.7% of aligned characters in a given position to be identical, or an alternative character for every six instances of the dominant character. Selecting lower entropies increases the risk of identifying sequencing errors as oligotypes while higher entropy thresholds would decrease the sensitivity of the method. We propose that applying a statistical method to determine a suitable threshold for each execution of the oligotyping procedure may provide a more robust and less subjective threshold criterion. The broken stick model, commonly used to predict the relative sizes of a randomly fragmented whole, may offer such a solution (Ramette and Buttigieg, 2014).

### DETECTING ‘RESOLVING’ OLIGOTYPES

We attempted to estimate the degree to which reads in an OTU have been distributed across oligotypes such that they may be used to differentiate between sites (i.e., ‘resolve’ sites based on their distributions) by calculating the average checkerboard (C) and togetherness (T) scores of each OTU-specific oligotype abundance matrix. We used C and T scores as they allowed us to screen for oligotypes with strong, presence-absence-based partitioning and aggregation among sites. This partitioning may be indicative of ecotype partitioning (i.e., competitive exclusion) as observed for other marine bacteria (e.g., Garczarek et al., 2007) and, if observed in repeated studies, may motivate taxon-targeted investigations to determine whether ecotype-level dynamics are in effect. Oligotypes which tend to co-occur at certain sites (e.g., **Figure 3A**, oligotypes TGT and -C at sites HGI–HGIII) may be indicative of subpopulations with similar levels of fitness in those locations. As an example, this screening approach revealed that oligotypes of OTU B177D, from the poorly characterized phylum Gemmatimonadetes, were associated with the highest average C and T scores (**Table 2** and **Figure 3B**). The Gemmatimonadetes have been observed in diverse environments, including soils and aquatic sediments, suggesting a diverse range of metabolic capacities in this phylum (DeBruyn et al., 2011). While confirmation is required, it is not unfounded to hypothesize that such metabolic plasticity may have translated into oligotype-level subpopulations colonizing HAUSGARTEN. Other oligotype matrices with high C and T scores include that of OTU C60MC (**Figure 3C**), classified as a representative of the Bacteroidetes. Apparent depth-related community composition changes within the Bacteroidetes have been observed in the Mediterranean (Díez-Vives et al., 2014), a trend somewhat echoed in our results where several oligotypes (CG, CA, and A-) were absent from shallower sites where others occurred (AT, AC, T, and G). One possible drawback of this approach is that C and T scores are binary measures and are not sensitive to differential abundance in oligotypes that are present in the same site. Thus this approach will not detect patterns which would, for example, indicate that one of a set of oligotypes appears to have greater fitness than others without leading to exclusion. To address this, the application of techniques dealing with abundance-based checkerboard and togetherness measures (Ulrich and Gotelli, 2010) may provide more informative results.

While outside the scope of this OTU-focused study, these results provide motivation to examine the higher-order taxa containing resolving oligotypes – alongside others found to have high C and T scores such as the Acidobacteria, Gamma-, Alpha-, and Deltaproteobacteria – through oligotyping. This will become an especially interesting undertaking as more next-generation sequencing datasets become available from the HAUSGARTEN LTER, enabling the detection of persistent oligotypes in the system and providing motivation for their further study. The natural consequence of confirming recurrent, site-resolving oligotypes is the formulation of hypotheses regarding the drivers of their differentiation in an effort to describe the microbial ecology at this scale.

### ENVIRONMENTALLY AND SPATIALLY STRUCTURED OLIGOTYPES

To complement the presence-absence-based checkerboard and togetherness analyses, we employed RDA – a multivariate form of multiple linear regression – to detect linear, abundance-based responses to environmental and spatial explanatory variables. Following our application of RDA and forward variable selection, we observed only seven of the 217 OTUs selected for analysis produced oligotype abundance matrices with greater than 50% explained variation. Indeed, a total of 55 models included at least one explanatory term in the model, while 162 models were trivial (i.e., ‘intercept-only’ models, featuring no explanatory terms). This result suggests that much of the oligotype-based microdiversity is not structured by the environmental or spatial factors measured; however, it may also imply that variables which are able to account for these responses have been overlooked. Additionally, we accept that our threshold for constrained variation is likely to be harsh for an ecological investigation: due to the sheer complexity of most ecosystems, it is not unusual to explain only a small fraction of the total variation in a response matrix (Cottenie, 2005). Nonetheless, we choose to err on the side of caution and report on oligotype matrices strongly structured by our explanatory variables. These results do show, however, that oligotype-level variation reveals patterns that are not evident at the OTU-level and that are related to environmental parameters.

In line with previous findings, water depth prominently featured in the models selected by our methods (**Table 3**). Several OTU-specific oligotypes appear to increase with depth (e.g., oligotype A of OTU D3V9F and oligotype A of OTU DTNEI; **Figures 4C,D** respectively) while others seem to have higher abundances in shallower regions (e.g., oligotype TC of OTU D3V9F; **Figure 4D**) or little response to varying depth (e.g., oligotype G of OTU ANOZB; **Figure 4G**). In several cases, oligotypes within a given OTU appear to show differential responses to depth (e.g., oligotypes A and C of OTU BGP4M and oligotypes TC and A of OTU D3V9F; **Figures 4C,D**, respectively). As discussed above and by Jacob et al. (2013), water depth is likely to act as a proxy variable for numerous depth-related parameters such as pressure or ecosystem composition (e.g., the community composition of larger organisms). Indeed, the negative correlation of depth with benthic phytodetritus concentrations (in our analysis, approximated by CPE concentrations) is reflected in the ordination of oligotypes derived from OTU BJCLU (**Figure 4A**). In this ordination, oligotype TGT appears at shallower sites with higher CPE concentrations, whereas other oligotypes appear to favor deeper sites with lower CPE concentrations. Thus, the prominence of depth as an explanatory variable in the RDA models above is unsurprising, but its exact relevance to the oligotypes derived from each OTU analyzed is more difficult to interpret. This provides motivation to design future sampling procedures that would capture a broader suite of depth-related contextual variables in aid of more precise characterization of bacterial community responses across taxonomic scales. Sampling during a natural perturbation which would decouple environmental factors that co-vary with depth (and are thus likely to confound one another in subsequent analyses) may also offer a particularly valuable opportunity to isolate their effects. Additional factors such as porosity, which has been observed to co-vary with benthic community structure in other Arctic sediments (Hamdan et al., 2013), and pigment concentration (partially indicative of energy availability in this system and likely associated with the presence of sea ice) are also linked to a bathymetric gradient; however, were not observed to be highly collinear with water depth. Thus, models such as that of OTU AHWYC (**Table 3** and **Figure 4F**) are important inasmuch as they are likely to reflect alternate ecological dynamics, worthy of pursuing in subsequent sampling designs. It is tempting to speculate that oligotypes with differential responses to variables such as depth and CPE concentration represent potential ecotypes. For example, based on their occurrence profiles and ordination by RDA, it may be hypothesized that organisms represented by the TGT oligotype of OTU BJCLU (**Figure 4A**) favor conditions where labile food sources are available (i.e., higher CPE concentrations), while those represented by oligotypes TCC and A are adapted to feeding on more recalcitrant compounds. A similar assertion may be made for oligotypes GG and A derived from OTU AHWYC (**Figure 4F**).

### OLIGOTYPE–OLIGOTYPE ASSOCIATIONS

Our network analysis of oligotype associations based on Whittaker’s IA revealed a large CC with scale-free properties, a trait that is frequently observed in biological and ecological networks, and several much smaller components (**Figure 5**). The variety of taxa and abundance classes which shared associations in the network and the MCL clusters derived from it (**Figure 6**) is a simple, but informative, result: oligotype associations cross taxonomic boundaries and abundance classes. This implies that oligotype-level variation reveals heretofore uninvestigated sub-OTU co-occurrence patterns that represent, for example, candidate bacterial guilds. Should these associations be validated with independent data (e.g., repeated sampling and sequencing of these HAUSGARTEN sites), they would provide motivation for targeted studies investigating specific sub-OTU microbial interactions. Additionally, the variation of consistently observed, closely associated oligotypes provides a reference against which one is able to identify which contextual parameters are of relevance to the microbial ecology of this rapidly changing ecosystem.

As a final note, we observed several nodes with high degree (≥25), but which corresponded to oligotypes with low abundance (≤5 reads). While true association cannot be ruled out, caution must be exercised in interpreting the associations of ‘rare’ oligotypes. While we did choose to remove absolute singletons (i.e., oligotypes which only had one read in the entire dataset), we did not use the oligotyping software’s parameters to restrict output based on the various abundance measures offered. While this may result in oligotypes generated from sequencing errors contaminating our results, it also prevents false negatives. As stated above, we suggest that the validation of oligotype occurrence through repeated sampling is a more tenable solution to this issue than arguments for or against a given, arbitrary threshold, which may have unpredictable effects on the analysis of count data (as shown in e.g., Gobet et al., 2010).

## CONCLUSION

This study adds both to the characterization of the bacterial benthos present at the HAUSGARTEN LTER and to the exploration of oligotyping as a methodology to detect heretofore undescribed bacterial microdiversity and ecology. Our results largely confirm previous observations linking responses in microbial community structure to water depth; however, they reveal a finer-grained response that can be both a source and target for new ecological hypotheses. Indeed, oligotypes from within a single OTU were observed to show differential occurrence across sites, respond differently to the explanatory variables analyzed, and associate with oligotypes derived from other OTUs. While work remains to be done in refining this approach and standardizing its application, oligotyping offers a readily applicable means to explore patterns in microbial microdiversity. Sequencing-enabled LTERs and Genomic Observatories (Davies et al., 2012, 2014) are uniquely positioned to evaluate oligotyping and similar methods through repeated sampling and validation and, in the process, have the opportunity to identify distinct microbial subpopulations and ecotypes central to their study site. The value of this capability is especially pronounced in regions undergoing rapid change, where a grasp of microbial responses at fine granularity is desirable.

## Conflict of Interest Statement

The authors declare that the research was conducted in the absence of any commercial or financial relationships that could be construed as a potential conflict of interest.
